# Association of Body Shape Index (ABSI) with Hand Grip Strength

**DOI:** 10.3390/ijerph17186797

**Published:** 2020-09-17

**Authors:** Nir Y. Krakauer, Jesse C. Krakauer

**Affiliations:** 1 Department of Civil Engineering, City College of New York, New York, NY 10031, USA; 2 Associated Physicians/Endocrinology, Berkley, MI 48072, USA; jckrakauer@gmail.com

**Keywords:** grip strength, sarcopenia, frailty, risk assessment, anthropometry, body shape

## Abstract

Hand grip is a leading measure of muscle strength and general health, yet its association with body shape is not well characterized. Here, we examine correlations between grip strength, a body shape index (ABSI), and body mass index (BMI) in the 2011–2014 United States National Health and Nutrition Examination Survey cohorts. Grip strength was found to correlate negatively with ABSI (though positively with BMI), suggesting that those with a more central body profile tend to be weaker than others with the same weight. Individuals with low grip strength, as well as those with high ABSI, were more likely to die during follow up, whereas there was no association of BMI with mortality hazard. Transforming the grip strength, ABSI, and BMI by taking their logarithm prior to standardization did not meaningfully change the associations seen. These findings suggest that combining anthropometrics (ABSI, BMI) with grip strength may better identify individual mortality hazard in research studies and clinical practice.

## 1. Introduction

Indicators of individual health that can be determined conveniently and at low cost [1] continue to play a central role in epidemiological and clinical assessment of the chronic and degenerative conditions that cause extensive morbidity and mortality [2]. Here, we consider the association within a population between two such measures of health, hand grip strength and a body shape index (ABSI), and compare their value as predictors of mortality hazard.

Grip strength can be measured easily with a portable dynamometer, and provides the most common single diagnostic measure of muscle strength and performance [3,4]. Grip strength is a marker of health, and low values have been found to predict fractures, disability, type 2 diabetes, hospital outcomes, all-cause mortality, cardiovascular and cancer death, and cardiovascular disease [5,6,7,8,9,10,11,12]. Longitudinal decline in grip strength for an individual is also a strong risk factor for adverse outcomes [13]. A recent review posits that grip strength is an umbrella assessment of the body systems that contribute to strength capacity and a measure of muscle strength that is representative of overall health status, and recommends including grip strength measurement in routine health assessments in clinical and epidemiological settings [14]. In healthy people, grip strength varies systematically with age and sex, and age and sex specific means and standard deviations have been published from different countries [15,16,17].

Grip strength is part of current definitions of sarcopenia, a construct which features low muscle mass combined with evidence of lack of strength (such as weak grip) [18,19]. Sarcopenia has traditionally been considered a manifestation of aging [20], and its progression is accelerated by comorbidities such as cancer, kidney disease, diabetes, and peripheral artery disease, as well as by poor nutrition and physical inactivity [21]. Sarcopenia is a strong risk factor for cardiometabolic and all-cause mortality as well as disability [22,23], and can to some extent be reversed with load-bearing exercise and dietary interventions [24,25] that can increase muscle mass and strength [26]. Low grip strength is also a marker of frailty [27] and malnutrition [28]. Although most prospective studies that quantified the impact of low grip strength on mortality risk have been of older adults [29], a number of large studies have also found higher risk associated with low grip strength measured at adolescence through middle age, for example in Swedish conscripts [30], nearly half a million participants in the British Biobank [31], and the multinational Prospective Urban-Rural Epidemiology (PURE) cohort [32]. Grip strength in youth also shows a strongly positive association with bone mineral content, which in turn has a strong inverse association with future fracture risk [33]. The genomics of muscle weakness are a subject of current investigations, and selected loci associated with lean tissue mass contribute to variability in effects of weight loss diets [34,35].

Sarcopenia, as well as cardiometabolic risk factors, is known to be associated with high waist circumference (WC) [23]. ABSI normalizes WC to body size (weight and height), analogous to body mass index (BMI) which normalizes weight (W) for height (H). ABSI is statistically independent of BMI, whereas variability in non-normalized WC is mostly explainable by BMI [36,37]. While the association of grip strength specifically with ABSI has not been studied in detail, there has been some research on association of muscle strength and sarcopenia with ABSI. Tay et al. [38] found that in functionally independent older adults, ABSI was higher in subjects with sarcopenia. Tay et al. [39] found that in community-dwelling older adults with mild cognitive impairment and mild to moderate Alzheimer’s disease, ABSI was not significantly different across categories of sarcopenia status. In both studies, the definition of sarcopenia included weak grip strength or slow gait speed as measures of strength, as well as low muscle mass from dual-energy X-ray absorptiometry (DEXA). In another study, ABSI was found to be inversely associated with DEXA-measured lean mass, independent of BMI [40]. Chung et al. [41] found that in a large national population sample, the Z-score of log-transformed ABSI was associated with both sarcopenic obesity and cardiovascular disease. Logarithmic transformation has been advocated for ABSI on the grounds that it might be expected to make the distribution of always-positive measures more normal [41,42,43,44].

This previous work shows low grip strength to be a well established risk factor for mortality and illness, while high ABSI is an emerging risk factor that has also been widely studied [45]. ABSI is derived from simple anthropometric measurements, making it a convenient clinical tool, and is known to be related to body composition, with high ABSI correlating to more abdominal fat and less limb lean mass. To what extent this difference in body composition also represents a difference in dynamic function, e.g., as measured by grip strength, has not been determined. The association with grip strength of ABSI, which quantifies body shape, can be compared and contrasted with that of BMI, a better established measure that quantifies body size. Additionally, how logarithmic transformation of ABSI and other always-positive quantities (which would include BMI and grip strength) affects their association with each other and with mortality hazard has not been studied. Considering these associations in a general population sample is a logical first step before considering how they might vary in subpopulations who may be at higher risk for sarcopenia or related conditions.

Based on these considerations, we examine the association between grip strength and ABSI, as well as BMI, in a national population examination sample. We also evaluate the relationship between grip strength and ABSI, on the one hand, and mortality over follow-up, on the other hand. Additionally, we consider whether log-transforming ABSI and the other anthropometric variables improves their associations with each other and with mortality.

## 2. Methods

### 2.1. Data

The United States (USA) National Health and Nutrition Examination Survey (NHANES) has been sampling the civilian non-institutionalized USA population since the 1970s using a cluster approach. Some groups of public health interest (children, the elderly, black and Mexican-American people) are deliberately oversampled. We analyzed the 2011–2012 and 2013–2014 NHANES cohorts, for which grip strength was measured using a Takei digital handgrip dynamometer (Takei Scientific Instruments, Shinagawa-Ku, Tokyo, Japan) [46]. A trained examiner explained and demonstrated the protocol to the participant, adjusted the dynamometer to the participant’s hand size, and asked the participant to squeeze the dynamometer for a practice trial. After the practice, the participant was asked to use one hand to squeeze the dynamometer as hard as possible. The test was then repeated for the other hand. Each hand was tested three times, alternating hands between trials, with a 60-s rest between measurements on the same hand [47]. We used the combined grip strength, defined as the sum of the largest readings from each hand. This was not available for participants only measured on one hand, who were therefore excluded from our analysis. Mortality outcomes for adult subjects were available from the National Center for Health Statistics through 2015 (1–5 years of follow-up, median: 3 years). We analyzed NHANES 2011–2014 data for all nonpregnant adults (age 18 and over) with the required measurements (including weight, WC, and grip strength) and mortality follow-up.

The protocol for NHANES has been approved by the National Center for Health Statistics Research Ethics Review Board as consistent with the Declaration of Helsinki. Ethics approval was not needed for the current study because only anonymized, public-use data from NHANES (https://www.cdc.gov/nchs/nhanes/index.htm) is employed.

### 2.2. Standardized Anthropometrics and Grip Strength

Anthropometric indices were calculated as follows [36,48]:

(1) $$\mathrm{BMI}\equiv W\cdot H^{-2},$$

(2) $$\mathrm{ABSI}\equiv \mathrm{WC}\cdot H^{5/6}\cdot W^{-2/3},$$

where W designates weight, H height, and WC waist circumference.

The anthropometric index values for the NHANES cohorts are converted to Z scores by subtracting the smoothed age and sex specific mean and dividing by the standard deviation [49], thus following the general formula:

(3) $$Z-\mathrm{score}\equiv \frac{\mathrm{value}-\mathrm{mean}}{\mathrm{std}.\mathrm{dev}.}.$$

Grip strength was converted to a Z score following the same process. These standardized values are referred to below as BMI, ABSI, Grip respectively. As an alternative, log-transformed Z scores were computed by taking the natural logarithm of BMI, ABSI, and grip strength before standardizing by the respective means and standard deviations of the logarithms [41]. These are referred to below as lBMI, lABSI, lGRIP respectively.

In all cases, means and variances (squared standard deviations) used in the Z score transformation were computed (for males and females separately) for each age, then smoothed across ages using a cubic spline with 4 degrees of freedom. An alternative specification of the smoothing cubic spline, where the number of degrees of freedom were set by minimizing a generalized cross-validation function, was also tried, and resulted in almost identical results for the correlations between anthropomorphic measures, grip strength, and mortality.

### 2.3. Relating Anthropometrics, Grip Strength, And Mortality Hazard

Grip strength Z score was regressed against BMI and ABSI Z scores as potential linear or nonlinear predictors and visualized using scatterplots. The nonlinear model used was a locally weighted polynomial (LOESS) smoother [50], which is widely used for visualizing nonparametric associations in scatterplot data because it combines the simplicity of linear regression with the flexibility of general nonlinear regression [51]. To better understand the relationship between BMI, ABSI, and grip strength in the NHANES population, we also show and discuss the correlation coefficients between them. This was repeated for the Z scores of the log-transformed BMI, ABSI, and grip strength.

The considered mortality predictors in Cox proportional hazard modeling [52] were the anthropometric index and muscle strength Z scores (BMI, ABSI, Grip) or the Z scores of their logarithms (lBMI, lABSI, lGrip). In line with previous analyses of NHANES [49,53], each mortality prediction model also included sex. Ethnicity was not included as a predictor since preliminary investigation showed that it did not have a significant association with mortality. Age was implicitly included, being the timescale in the Cox model [36]. The model for BMI as a linear predictor, for example, took the following form:

(4) $$\lambda_{i}=\lambda_{0}\left(t_{i}\right)\exp\left(\beta_{0}s_{i}+\beta_{1}{\mathrm{BMI}}_{i}\right),$$

where the subscript *i* refers to any one of the participants, λ is the estimated mortality hazard, $\lambda_{0}$ is the baseline mortality hazard that is an arbitrary function of age *t*, *s* is a binary variable designating sex as male or female, BMI is BMI Z score, and $\beta_{0},\beta_{1}$ are fitted coefficients. A model with BMI as a nonlinear predictor could use a penalized spline basis to parameterize a more complicated function of BMI [36,54]; however, it turned out that nonlinear terms in the fitted spline (for the model with BMI as well as analogous ones with ABSI and Grip) were not different from zero with low *p* value, so only results for linear models such as Equation (4) are tabulated below.

As in Krakauer and Krakauer [49], the main measure of relative model performance was Akaike information criterion (AIC) difference score, $\Delta_{i}.$ For the best-performing model (with lowest AIC) $\Delta_{i}=0,$ while other models have positive $\Delta_{i}$ [55]. $\Delta_{i}>6$ indicated models that perform significantly worse than the best-performing model (at the 95% confidence level) as mortality predictors for the sampled population [56]. We also calculated $R^{2}$ values, denoting the proportion of variation in mortality explained by the predictors of each model, so that higher $R^{2}$ suggests a model with greater explanatory power [57]. Another measure of model mortality-prediction performance considered was concordance (*C*), defined as the fraction of pairs of individuals in the sample for which the one modeled to be at greater risk actually died sooner [54]. Concordance ranges from 0 to 1, with 0.5 the expected value for models with no skill and higher values indicating models that are more skillful at explaining variation in survival.

All computations were carried out in R software. Scatterplot LOESS smoothing used the loess function in the stats package. Smoothing cubic splines for computing Z scores used the pspline package. Proportional hazard modeling used the survival package [54].

## 3. Results

Out of 19931 NHANES 2011-2014 participants, 11977 were adults, and 9803 met the criteria to be included in the current analysis. Of these 9803 individuals, 50% were male, and the median age at examination was 46 (interquartile range: 31–61). Ethnicity was coded as 12% Mexican, 9% Other Hispanic, 40% White, 23% Black, 12% Asian, 3% Other. Median height was 174 cm for males and 161 cm for females, median weight was 83 kg for males and 72 kg for females, and median BMI was 27.3 for males and 27.9 for females. There were 256 deaths over the follow-up period.

Table 1 shows the correlation coefficients among ABSI, BMI, and Grip Z scores, without and with logarithmic transformation. ABSI was uncorrelated with BMI. With grip strength, ABSI had a negative correlation and BMI had a positive correlation, both of similar magnitude (around 0.2), implying that people with high BMI and low ABSI tended to have stronger grip. The Z scores after transformation were very highly correlated with the respective non-transformed measures (r>0.95), and had very similar associations with each other as the non-transformed measures.

**Figure 1:**
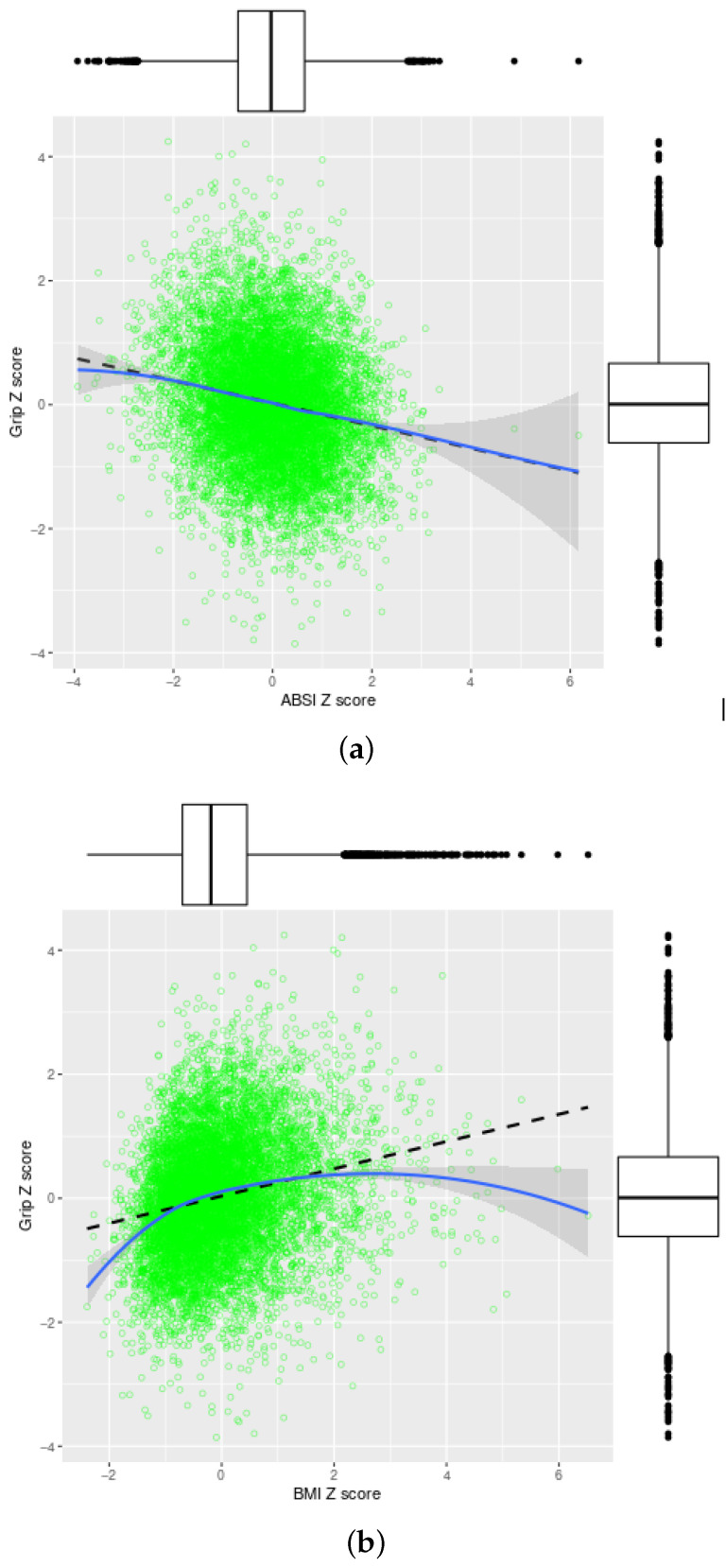
Grip strength versus (**a**) ABSI and (**b**) BMI Z score in NHANES 2011–2014. The dashed black line shows the least-squares linear fit. The solid blue line shows a local polynomial (LOESS) fit, with the shading indicating a 95% confidence interval for the fit. The axis box and whisker plots show the marginal distributions. ABSI = a body shape index, BMI = body mass index, Grip = hand grip strength.

Indeed, the association of Grip with ABSI is linear and inverse, with each standard deviation increase in ABSI associated with a 0.18±0.01 standard deviation decrease in grip strength (Figure 1a). The association of Grip with BMI is in the opposite direction, with some nonlinearity: at lower than average BMI, each standard deviation increase raises grip strength by as much as 0.6 standard deviations, but grip strength levels off as BMI rises in the morbid obesity range (Figure 1b).

ABSI was a significant linear predictor of mortality, while BMI was not. Grip strength was an even better linear predictor of mortality than ABSI. The log-transformed lABSI, lBMI, lGrip predictors had very similar skill for mortality prediction as the non-transformed ones (Table 2). Allowing potential nonlinear associations using penalized spline models showed that the association of ABSI and Grip with mortality hazard was best modeled as linear, while for BMI there was no significant nonlinear relationship either with mortality hazard.

**Figure 2:**
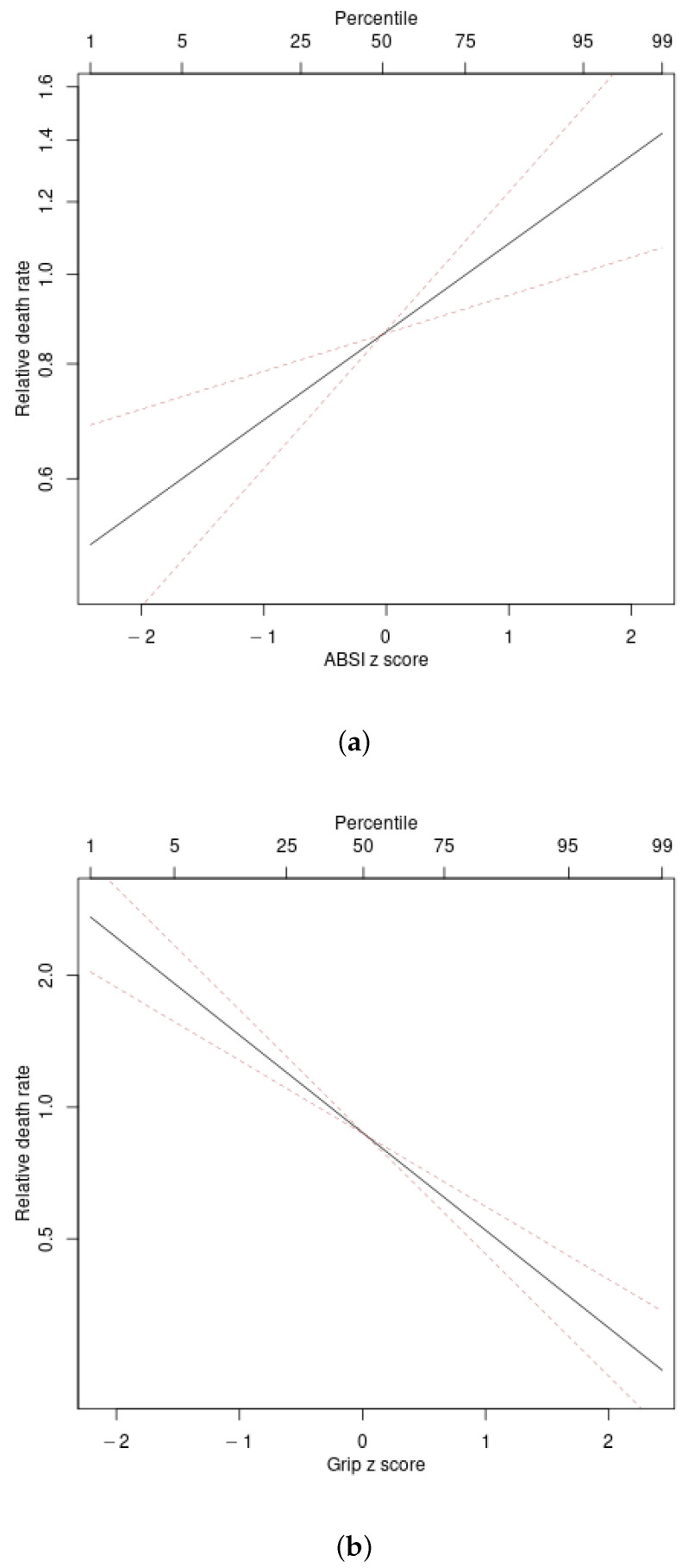
Estimated mortality hazard ratio in NHANES 2011–2014 as a linear functions of (**a**) ABSI, (**b**) Grip Z score. Dashed lines indicate 95% confidence intervals. Percentiles are based on the NHANES 2011–2014 cohort. ABSI = a body shape index, Grip = hand grip strength.

Figure 2 illustrates the associations found between ABSI and grip strength Z score and mortality hazard. Adjusting for age and sex, the logarithm of mortality hazard increases linearly with ABSI and decreases linearly with grip strength.

## 4. Discussion

### 4.1. Correlation of Grip Strength and Anthropometrics

The public access NHANES data analyzed here include both anthropometric and hand grip strength measurements in a national sample of near 10,000 people. Medium term mortality from 1–5 years of follow-up allowed for comparison of hazards for simultaneously determined transformations of the baseline measures. Hand grip can be seen directly reflecting pathophysiology with influence on longevity. Anthropometrics would be more indirectly determined by underlying pathophysiology. For example, high ABSI can be associated with loss of skeletal mass from the extremities in sarcopenia [40,41]. The current study is the first to describe the negative association of ABSI with grip strength, as well as finding a nonlinear (i.e., leveling off with morbid obesity) positive association of grip strength with BMI.

ABSI and BMI are conceptually related as allometric adjustments of, respectively, weight for height and WC for weight and height, and are meant as complementary rather than alternative measurements. The current analysis attests near zero correlation of BMI to ABSI Z scores, without or with log transformation. The statistical independence criteria here are consistent with the initial derivation of ABSI from a larger NHANES 1999–2004 population sample [36]. We suggest that applications of ABSI in other populations should include assessing the correlation with BMI. When nearly independent, it is appropriate to accept ABSI as a body shape index that complements body size as indicated by BMI.

After Z score transformation, the magnitude of the correlations of grip strength with ABSI and BMI was about 0.2, meaning that most variability in grip strength could not be accounted for by these simple anthropometrics. This correlation magnitude is similar to that previously found between ABSI and MS score [53], and manifests in the extensive scatter depicted in Figure 1. We tentatively interpret this relatively low correlation as indicating that grip strength on the one hand and ABSI and BMI on the other measure different, though overlapping, aspects of health, and may have differing metabolic and genetic correlates. Note that higher correlations of 0.7–0.8 have been reported between hand circumference and grip strength [58], and correlations between grip strength and knee extension strength, as a measure of lower body strength, are similarly high [59].

### 4.2. Associations with Mortality Hazard

The mortality hazard evaluation result is compatible with the relationship with mortality that has been previously described for grip strength. Mortality hazard for ABSI and BMI have also been previously described and connected with abdominal obesity as conceptualized by metabolic syndrome (MS). In fact, including ABSI in place of WC or BMI optimized mortality prediction with MS scoring [53].

The current data provide the first direct comparison of mortality hazard with grip strength together with ABSI and BMI. The current findings show that strength testing improves predictive power for mortality hazard beyond simple anthropometrics and thus provide support for its widespread inclusion in clinical assessment.

### 4.3. Logarithmic Transformation for Anthropometrics and Grip Strength

The current analysis is also the first to compare the inter-correlation and predictive power for mortality of Z scores based on the original definition of ABSI (as well as BMI and grip strength) versus ones based on the logarithm of ABSI (or the other measures). In addition to being advocated for ABSI, such log transformation has been considered for regression analyses for variables such as BMI to reduce skewness and improve normality [60,61,62,63]. We found that such transformations do not improve predictive power. Especially for ABSI, the range of values is quite narrow, so that logarithmic transformation typically has little effect on the Z scores. Even for BMI and grip strength, where the range of population values is larger and the distribution is thus more skewed to the right, logarithmic transformation was found not to improve association with mortality hazard. Based on our findings, we postulate that ABSI Z score with or without logarithmic transformation will have very similar associations with adverse outcomes, including mortality. This is subject to verification with other cohorts and outcomes.

### 4.4. Strengths and Limitations

Strengths of the current study include using a large nationally representative population sample that enabled fairly precise determination of the associations between BMI, ABSI, and grip strength. Limitations include the limited number of deaths due to short follow-up (which may explain lack of significant association of mortality hazard with BMI in this study, unlike other studies which show a U-shaped nonlinear association), lack of DEXA body composition (which is available for some earlier NHANES cohorts [64,65]) to correlate with anthropometrics and grip strength, and lack of hip measurements, which form another potential anthropometric mortality predictor [49] and which could also be investigated for associations with grip strength and sarcopenia. We also considered a general adult population, and did not specifically quantify interactions of the associations we found with risk factors and comorbidities; additional studies could help verify the generalizability of our results to the disproportionately older populations with acute or chronic conditions who are most vulnerable to sarcopenia and frailty.

## 5. Conclusions

We demonstrate the expected statistical independence of ABSI and BMI and the predictive value of ABSI for grip strength in a well characterized population sample. The relationships hold after Z score transformations for sex and age, with little advantage accruing from log transforming the data. Subsequent mortality was most strongly associated with low grip strength, as well as with elevated ABSI. Combining basic anthropometrics with grip strength may better identify meaningful individual mortality hazard in research studies and clinical practice.

## Figures and Tables

**Table 1:** Correlations of body and strength measures.

	ABSI	BMI	Grip	lABSI	lBMI	lGrip
ABSI		0.00	−0.19	1.00	0.00	−0.20
BMI			0.21	0.00	0.99	0.20
Grip				−0.18	0.23	0.96
lABSI					0.00	−0.19
lBMI						0.22
lGrip						

Correlation coefficients for body and strength measures (Z scores relative to age-, race-, and sex-specific means, without or with logarithmic transformation) among NHANES 2011–2014 nonpregnant adults. ABSI = a body shape index, BMI = body mass index, Grip = hand grip strength. Prefixed l refers to logarithmic transformation prior to computing Z score.

**Table 2:** Mortality hazard association with body and strength measures.

Predictor	Hazard Ratio per SD Increase	$\Delta_{i}$	$R^{2}$	*C*
ABSI	1.25 (1.10–1.41)	48.6	0.055	0.594
BMI	1.04 (0.91–1.18)	60.2	0.028	0.554
Grip	0.60 (0.53–0.68)	0	0.165	0.660
lABSI	1.25 (1.10–1.42)	48.4	0.055	0.594
lBMI	1.01 (0.88–1.14)	60.4	0.027	0.554
lGrip	0.64 (0.58–0.71)	2.6	0.159	0.659
None		58.4	0.027	0.553

Results of Cox proportional hazard modeling for mortality risk in NHANES 2011-2014 with BMI, ABSI, or Grip Z scores (or the Z scores of their log transformations, lBMI, lABSI, lGRIP) taken as linear predictors. All models also included sex as a predictor. Ranges in parentheses are 95% confidence intervals for the hazard ratio. ABSI = a body shape index, BMI = body mass index, Grip = hand grip strength. Prefixed l refers to logarithmic transformation. SD = standard deviation; $\Delta_{i}$ = Akaike information criterion score difference relative to the best performing model shown (see Methods for details); $R^{2}$ = measure of explained variation; *C* = concordance.
